# Supplementary material for: Mind the Wound!—Fruit Injury Ranks Higher than, and Interacts with, Heterospecific Cues for Drosophila suzukii Oviposition
Source: Insects. 2021 May 9;12(5):424. doi: 10.3390/insects12050424 (PMC8151711; doi:10.3390/insects12050424)
Supplement: Supplementary file 1 [file insects-12-00424-s001.zip › insects-1194480-supplementary.pdf]

Treatmer	Replicat	Summe	SummeF	SummeX
A	A31	8	5	3
A	A35	8	4	4
A	A4	9	1	8
A	A6	9	8	1
A	A1	10	0	10
A	A23	13	9	4
A	A30	13	2	11
A	A27	15	15	0
A	A32	16	15	1
A	A3	18	15	3
A	A2	23	15	8
A	A5	23	13	10
A	A29	27	17	10
A	A34	27	26	1
A	A22	30	12	18
A	A24	30	26	4
A	A26	30	27	3
A	A28	32	29	3
A	A7	33	20	13
A	A25	33	25	8
A	A33	33	23	10
B	B5	3	1	2
B	B23	3	3	0
B	B1	4	0	4
B	B34	8	2	6
B	B4	10	5	5
B	B27	10	6	4
B	B29	14	6	8
B	B3	16	12	4
B	B6	17	13	4
B	B28	19	8	11
B	B24	21	15	6
B	B26	21	17	4
B	B25	24	24	0
B	B7	25	15	10
B	B32	25	24	1
B	B35	27	15	12
B	B31	30	23	7
B	B2	33	25	8
B	B22	38	34	4
B	B30	40	29	11
B	B33	44	20	24
C	C31	6	5	1
C	C6	9	9	0
C	C5	10	8	2
C	C7	12	12	0
C	C29	13	11	2
C	C33	16	12	4
C	C4	17	17	0
C	C30	20	18	2
C	C32	23	19	4
C	C1	24	16	8
C	C3	25	19	6
C	C22	26	11	15
C	C27	26	21	5
C	C23	27	15	12

C	C2	28	23	5
C	C36	28	18	10
C	C24	29	18	11
C	C28	32	24	8
C	C34	39	29	10
C	C26	41	33	8
C	C25	42	23	19
C	C35	56	37	19
D	D1	49	34	15
D	D2	42	19	23
D	D3	47	34	13
D	D4	34	5	29
D	D5	47	18	29
D	D6	34	19	15
D	D7	45	32	13
D	D8	49	28	21
D	D9	34	32	2
D	D10	68	53	15
D	D11	49	33	16
D	D12	49	10	39
D	D13	64	35	29
D	D14	42	23	19
D	D15	50	39	11
D	D16	46	33	13
D	D17	61	42	19
D	D18	66	38	28
D	D19	54	16	38
D	D20	47	33	14
D	D21	66	49	17
